# Health inequalities between employed and unemployed in northern Sweden: a decomposition analysis of social determinants for mental health

**DOI:** 10.1186/s12939-018-0773-5

**Published:** 2018-05-16

**Authors:** Anna Brydsten, Anne Hammarström, Miguel San Sebastian

**Affiliations:** 1 Department of Public Health Sciences, Stockholm University, SE-106 91 Stockholm, Sweden; 2 Department of Public Health and Caring Sciences, Public Health Unit, Uppsala University, SE-751 22 Uppsala, Sweden; 3 Department of Public Health and Clinical Medicine, Epidemiology and Global Health Unit, Umeå University, SE-901 85 Umeå, Sweden

**Keywords:** Social determinants of health inequality; unemployment, Life course, Northern Sweden, Oaxaca decomposition analysis, Mental health

## Abstract

**Background:**

Even though population health is strongly influenced by employment and working conditions, public health research has to a lesser extent explored the social determinants of health inequalities between people in different positions on the labour market, and whether these social determinants vary across the life course. This study analyses mental health inequalities between unemployed and employed in three age groups (youth, adulthood and mid-life), and identifies the extent to which social determinants explain the mental health gap between employed and unemployed in northern Sweden.

**Methods:**

The *Health on Equal Terms* survey of 2014 was used, with self-reported employment (unemployed or employed) as exposure and the *General Health Questionnaire* (GHQ-12) as mental health outcome. The social determinants of health inequalities were grouped into four dimensions: *socioeconomic status, economic resources, social network* and *trust in institutional systems*. The non-linear Oaxaca decomposition analysis was applied, stratified by gender and age groups.

**Results:**

Mental health inequality was found in all age groups among women and men (difference in GHQ varying between 0.12 and 0.20). The decomposition analysis showed that the social determinants included in the model accounted for 43–51% of the inequalities among youths, 42–98% of the inequalities among adults and 60–65% among middle-aged. The main contributing factors were shown to vary between age groups: cash margin (among youths and middle-aged men), financial strain (among adults and middle-aged women), income (among men in adulthood), along with trust in others (all age groups), practical support (young women) and social support (middle-aged men); stressing how the social determinants of health inequalities vary across the life course.

**Conclusions:**

The health gap between employed and unemployed was explained by the difference in access to economic and social resources, and to a smaller extent in the trust in the institutional systems. Findings from this study corroborate that much of the mental health inequality in the Swedish labour market is socially and politically produced and potentially avoidable. Greater attention from researchers, policy makers on unemployment and public health should be devoted to the social and economic deprivation of unemployment from a life course perspective to prevent mental health inequality.

## Background

Health inequalities between employed and unemployed have been found in Sweden and other European countries – in different labour market groups, across time, in different macroeconomic settings and between women and men [1–10]. However, few studies have elaborated on the issue of disentangling health inequalities and identifying the determinants of the health gap between the employed and unemployed. The present study seeks to contribute by focusing on the social determinants of health inequalities in self-reported mental health between employed and unemployed in three different age groups in northern Sweden.

### Unemployment in a Swedish context

The present study unfolds in the wake of the financial crisis in 2008, in a welfare state actively trying to decrease health inequities among citizens [11]. The national unemployment rate is currently higher than before the crisis (7.9% of the working age population compared to 5–6%), men have higher unemployment than women, and youths (15–24 years) have the most difficulties finding work (22.9% in unemployment) [12, 13]. Sweden is characterised as a worker-friendly country with universalism, solidarity and de-commodification of social and economic equality between its citizens [14]. It is a welfare model constructed to reduce inequities between classes through benefits such as social insurance, parental leave benefits, state-funded pension, free higher education tuition, free health care, and day-care, child allowance and school lunches for all children. It is a costly burden of social services and transfers funded by public taxes, and thus with high demands on all citizens to contribute by working full-time and paying taxes [14]. For those in unemployment, i.e. involuntary lack of paid work, the Swedish state has a de-commodification system for income loss and self-worth by cash programmes to help unemployed to find ways back into work (by passive and active labour market matching) [9]. However, during the last few decades the Swedish welfare state has been undergoing major changes, in accordance with macroeconomic change in the world economy, leading to downsizing in income protection and other welfare benefits [11]. In fact, when the cost of social benefits increased during the economic crises and unemployment rates grew in the 1990s and in 2008, cuts were made in the state-funded unemployment benefits [9, 10], making those in unemployment a particularly economic vulnerable group. Recent reports have described an alarming development with increasing socioeconomic inequalities, child poverty and cuts in the state-funded social security systems [15–18]. Even though Sweden still has generous welfare policies and strong focus on equity in health [11], the system has not been able to reduce the health inequities between social classes in society [19].

### Unemployment and social determinants of health

In this study unemployment is conceptualised as a social and material exclusion from the normative wage working society [20]. It is a stressful life event regardless of age and may affect health through embodied stress and emotional strain [21]. Previous research has shown a clear relationship between unemployment and concurrent health problems, both with mental health illness such as general poor mental health, anxiety and depression, and with physical health outcomes such as high blood pressure and cortisol levels, heart diseases and mortality [4, 5, 22]. However, studies on health inequalities have mainly focused on social determinants such as income, social status, gender and place of residence [23], overlooking work and unemployment as one of the most important social determinants of health inequality [20]. A direct consequence of unemployment can be poverty and economic deprivation, where income loss is likely to restrict life choices, the ability to plan ahead and the feeling of control [24]. Financial strain and low cash margin also tend to increase stress and impair mental health among unemployed [25]. Economic deprivation may also act as a moderating factor through socioeconomic circumstances of low education, working conditions of manual work and low income, leading to higher risk of unemployment and psychological distress. Beyond the income loss, unemployment is also a deprivation of psychosocial aspects of work, such as feeling of belonging, self-identity, social interaction and integration in society, which are important protective factors for mental health [26]. People in employment spend a lot of time at the workplace and it becomes an essential part of the social activities of everyday life. Hence, unemployed people tend to be less active and more socially isolated. It may partly be due to the lack of income, and thus the ability to be a consumer in a society organised around consumption of goods and services. It may also be related to loss of status and shame at not being a part of the working society [27]. However, access to social buffers can dampen the loss of work-related social isolation by increasing the individual’s ability to cope with the emotional strain of unemployment, such as lack of social interaction with colleagues. This might be the social network of family and friends, providing someone to talk to or practical support [28, 29]. Alternative role identities beyond the working identity, such as social participation, can also act as a social buffer for ill-health related to unemployment [27]. Participation in social movements and public debate may lead to better social, mental and physical health regardless of employment position [30], and thus challenge the normative idea of work as a critical source of individual growth, self-fulfilment, social recognition and status [27].

Another important aspect relates to how individual health status can be shaped by the macro- and meso-level systems of society, such as the organisation of work and the labour market, but also the availability of systems such as income protection and access to health care [31, 32]. Active labour market policies have been shown to moderate the impact of health hazards during unemployment [33–35], to facilitate the ways back to re-employment [36] and reduce the worries and fear regarding risk of unemployment among those in employment [37]. In fact, psychological and economic research has shown that job insecurities and fear of unemployment affect well-being and life satisfaction [37], implying that it is not just the actual implications of generous social policies that can reduce ill-health but also that subjective trust and confidence in institutionalised social benefits might have health-buffering implications. In contrast, those in highest need of health care and social security may also be those at highest risk of unemployment, with low resources for individual economic and social protection, leading to a decline in trust in the ability of welfare systems to care for the citizens. Thus, the social insurance may modify the health implications of unemployment, while preserving the inequalities in health in the population [38].

### Unemployment and ill-health in different phases of life

Sociological and epidemiological life-course researchers recognise three main stages of life corresponding to the position on the labour market, social and psychological development and the social and material circumstances characterising each life phase [39, 40]. The first phase is *young adulthood* (late teens to mid-twenties). It is typically illustrated as the stage in life when young people are expected to enter adult society by normative age-specific events, such as leaving school, entering the workforce, leaving the parental home and engaging in long-term relationships. Even though none of these social roles transitions is required for the achievement of adult status, together they mark the adult stage of the life course development [41] and unemployment may delay some of these age-normative events. This is sometimes referred to as ‘delayed adulthood’ [41], with implications for the health status of young unemployed people. On the other hand, youths tend to have a relatively low financial burdens compared to people during other stages of life and they often share the experience of instability in labour market attachment with their peers. Thus, the stigma related to unemployment may be lower among youths. The second phase is *adulthood* (late twenties to 40), often illustrated as the ‘settling down’ phase which often involves an increased investment in work, family and friends [40]. It is the time in life between the intensive phase of becoming an adult and before the more critical health problems accruing in mid-life, thus a phase with typically good physical health [40] but nevertheless high lifestyle stress. This is a time in life when many become parents, with higher demands for financial stability and when the social norm of employment is high, i.e. the social expectation of wage work is closely related to the feeling of belonging, identity and taking part in society. Unemployment during this phase may affect the emotional strain in relationships with family and friends, illustrated in the higher risk of divorce among unemployed [42]. The third and last phase in the labour market is *mid-life* (40 to 65 years), sometimes referred to as the time of the ‘empty nest’; children move from the parental home and the financial burden is lower. Most people have a relatively stable position in the labour market during this period of life. However, becoming unemployed during this phase is troubling due to age discrimination in the labour market. Becoming re-employed has been shown to be difficult, due to ideas about this group having difficulties adapting to new technology and changing society and cultures [43, 44], and thus with implications for health status [45]. This is also a time in life when age-related health problems start to become more frequent and when unemployment may be an even higher contributor to health inequalities.

In sum, previous studies within the field are limited, typically focusing on the relationship between unemployment and health, rather than on which contributing factors can explain the differences in health [46]. There are also reasons to imagine that social and economic circumstances may differ across age groups [47], resulting in different factors influencing the health inequalities between employed and unemployed. The aim of the present study was to investigate the mental health inequalities between employed and unemployed across three age groups of youth, adulthood and mid-life in northern Sweden and to identify to what extent social determinants could explain the mental health gap between the two groups.

## Methods

### Sample and procedure

The current study was conducted on the *Health on Equal Terms* survey. This is a Swedish public health survey conducted every four years since 2004. The survey is administered by the Public Health Agency of Sweden in collaboration with the four northern county councils and Statistics Sweden [48]. The survey covers areas such as health and well-being, contact with health care, living habits, work and occupation, and social relationships. Register data from Statistics Sweden were also collected for all participants about areas such as education, income and civil status. In the four northern counties of Sweden (Västernorrland, Jämtland, Västerbotten and Norrbotten) the last data collection was conducted in 2014, comprising 708,641 inhabitants in the age 16 and 84. From this population, a randomised sample was selected, stratified by sex, age and municipality. The response rate was approximately 50% (*n* = 25,667). In the present study the effective sample was *n* = 10,407 individuals (of whom 7 .7% were unemployed), due to exclusion of people over age 65 (*n* = 8888), and people outside the labour market, such as students, on sick leave, on parental leave, in labour market measures (*n* = 4001). Individuals with missing values in some of the variables also became missing in the final analysis (*n* = 2371), most frequent in financial strain, cash margin and support. However, no systematic pattern was found in the missing values.

### Measurements

Psychological distress, measured by the twelve-item version of the General Health Questionnaire (GHQ-12), was used as *mental health* outcome. It is a widely used self-administered screening instrument developed for non-psychotic mental illness, typically depressiveness and anxiety [49]. The participants were asked if they had experienced different symptoms or behaviour during the last few weeks, such as being able to concentrate and enjoying day-to-day activities, feeling unhappy, depressed, and capable of making decisions. Each item assesses the severity of the symptoms with a four-point scale as ‘less than usual’, ‘no more than usual’, ‘rather more than usual’ or ‘much more than usual’. The items were recoded into more or less severity and then summed up into an index with the range 0–12, and dichotomised by three or more symptoms, commonly used in surveys to indicate psychological distress [49].

The variable unemployment was self-reported, capturing the subjective experience of involuntary lack of paid work. Participants’ current labour market position was dichotomised into unemployed and employed (including self-employed), excluding those in labour market measures, students, those on parental leave and different forms of sick listing in order to make as homogenous comparison groups as possible. Different dichotomising of labour market position was tested, such as unemployed or in labour market measure and outside the labour market, with similar results.

Age was categorised into three groups: *youth* between age 16 and 25, *adults* between age 26 and 39 and *middle-aged* between age 40 and 65. Age groups were categorised in accordance with a sociological and epidemiological life course framework of different stages in life (see above).

### Determinants of health inequities

Determinants of health inequalities were divided into four different domains: *socioeconomic status*, *economic resources*, *social network* and trust in *welfare institutions*. Because the health consequences of unemployment runs across all levels of the society, the variables education, civil status and occupational class measured socioeconomic status. Highest level of *education* was based on register data collected by Statistics Sweden 2013, and coded into high and low level of education. Low education included compulsory education and 2 years’ upper-secondary education without eligibility for higher education, and high education as 3 years of secondary education until PhD degree. Participants’ *civil status* was based on both register and questionnaire data, to capture the common living arrangement of non-married cohabitation in Sweden. Statistics Sweden’s register data of civil status registered in 2014 were used as married, unmarried, divorced and widow, while the self-reported questions were used for cohabiting living arrangement. The civil status variables were coded into married/cohabiting and unmarried/not cohabiting (including divorced and wido*w*/widower). *Occupational class* was, unlike the self-reported unemployment, based on Statistics Sweden’s socioeconomic classification last registered employment [50] and coded into blue-collar worker (unskilled manual workers and skilled manual workers) and white-collar workers (assistant non-manual, intermediate non-manual, self-employed and other).

Economic resources were measured by three variables: income, cash margin and financial strain. *Income* was based on the individual disposable income of the register-based debt and assessed (such as earnings from employment and capital, housing and child benefits, student loan, repayments of student loan and alimony) and coded into five quintiles. Participants’ *cash margin* was captured by the question ‘Would you be able to get hold of 15 000 SEK in one week for an unforeseen situation?’ with the response option as yes or no. *Financial strain* was captured by the question ‘During the past 12 months, have you had difficulties managing your ongoing expenses, such as food, rent and bills?’ which was dichotomised into yes or no.

The social dimension was measured by the subjective feeling of access to social network, divided into practical, social support and trust in others and social participation. *Practical support* was measured by the ability to get help in case of practical problems or illness and dichotomised into ‘always or most of the time’ and ‘never or not for the most parts’. *Social support* was measured by the question ‘Do you have someone to share your thoughts and inner feelings with?’, while *trust in others* was measured by the question ‘Do you think, in general, that one can trust most people?’ both with the response options yes or no. Social participation in leisure time activities was measured by the matrix question ‘Have you participated in some of the following activities during the last 12 months?’ with fourteen different options of activities. Two different variables of alternative roles were created: socially active and participation in sports events. The first one was measured by participation in a public event, such as night club or dancing, larger family gatherings and/or private party. The second one asked whether or not they had participated in public sport events. Both variables were dichotomised into active or none active. The remaining alternative roles were also tested, such as participation in leisure time study circle, labour market unions or other types of non-governmental organisations, public debates as well as cultural events such as theatre, movies and art exhibitions, but they were not included in the final analysis since they did not contribute to the health gap.

Two variables were constructed to capture the general belief in the welfare institutions. The matrix question was worded as ‘Do you trust the following institutions/politicians in society?’, and asked to rate on a five-point scale from ‘very high’, ‘rather high’, ‘rather low’, ‘very low’ and ‘no opinion’ for twelve different institutions. The first constructed variable, *confidence in unemployment benefits*, consists of attitudes towards the state-administered unemployment office and the income benefit. The variables were added and dichotomised into ‘high or very high trust’ and ‘low or very low trust’. Those with no opinion were coded as missing. The second variable, *confidence in health care***,** was elicited and coded in the same way as the variable above.

### Analysis

Frequencies of the characteristics for unemployed and employed were calculated for each determinant of health. Pearson’s chi-square was used to assess statistical differences between unemployed and employed within each of the age groups.

To address the aim of the study, a Blinder-Oaxaca decomposition analysis for non-linear regression models was applied [51], decomposing the mental health gap between unemployed and employed into three age groups (youths aged 16 to 25, adults 26 to 39 and middle-aged 40 to 65). The analysis was conducted in two steps and performed stratified for each age group and for genders. First, the difference in mental health was estimated, between the disadvantaged group (the unemployed) and the non-disadvantaged group (employed), presented as log odds. Then a decomposition was conducted to assess the magnitude of the explained and unexplained components along with a detailed decomposition to display each predictor contribution of the explained and unexplained association with the health inequalities. The *explained* component reflects the contribution of each social determinant to the difference in health gap between the groups, and is interpreted as the difference of characteristics between unemployed and employed that could explain some part of the total health gap [52]. The *unexplained* component captures the part of the health gap that remains unexplained. It can be interpreted as a discrimination or an unequal treatment of the unemployed against the employed (with the assumption that all determinants of health are included in the analysis) or uncertainty due to residuals [53]. Estimations were reported as log odds along with the share of the relative contribution of each determinant of health (presented as percentage of the total explained contribution) to the health inequalities. Calculations of confidence interval were presented as the significance at 5% level [52]. This approach has previously been applied within fields such as gender income-inequalities, health inequalities and socioeconomic gaps in health [53–55].

All the analyses were performed in Stata 13. Estimations were obtained with the Oaxaca command specifying the logit and pooled options. The logit option is the non-linear decomposition for binary dependent variables, providing a way to estimate both aggregated and detailed decompositions [51] while the pooled option applied a pooled model (as an additional variable) of both groups’ coefficients as the reference coefficient [56]. Independent variables, such as trust in school, social service, parliament, local politicians and trade unions, without contribution to the health gap (coefficients< 0.00) were excluded from the analysis. Ordinary Blinder-Oaxaca analysis and Fairlie decomposition [57] were also carried out, giving similar results.

## Results

Descriptive statistics are presented in Table 1, showing the pattern of characteristics between unemployed and employed, separately for each age group. The results show that those in unemployment, across all age groups, were more likely to be low educated, not cohabiting, blue-collar workers and have lower access to economic resources and social networks, compared to their employed counterparts. Among youths and adults no significant difference was found between unemployed and employed men and women, while men in middle age were more likely to be unemployed than women. Low trust in the health care system was more common among unemployed in adulthood and middle age, while trust in unemployment benefits was low in all ages regardless of labour market position. Altogether, unemployed in all life stages were more socially and materially disadvantaged than the employed.

**Table 1:** Descriptive characteristics of unemployed and employed in three age groups (% within unemployed and employed)

	Youth (age 16 to 25)					Adulthood (age 26 to 39)					Middle age (age 40 to 65)				
	*Unemployed*		*Employed*			*Unemployed*		*Employed*			*Unemployed*		*Employed*		
	%	n	%	n	p	%	n	%	n	P	%	n	%	n	p
Determinants															
Male gender	49.6	143	43.2	310		43.0	125	46.4	1677		57.6	234	47.3	3527	***
Low education	52.6	141	31.4	222	***	29.4	82	10.1	363	***	62.0	248	46.8	3481	***
Not cohabiting civil status	72.9	210	61.1	439	***	43.3	126	23.0	830	***	35.0	142	18.0	1430	***
Blue-collar occupational class	91.0	259	74.3	520	***	75.8	213	41.1	1452	***	67.4	263	39.1	2859	***
Lowest income quintile	81.5	233	56.8	407	***	43.5	123	9.1	328	***	21.7	87	3.9	292	***
Cash margin	49.7	143	18.4	132	***	50.2	146	14.1	508	***	41.4	168	9.6	714	***
Financial strain	33.2	95	18.6	133	***	46.1	134	13.4	482	***	29.95	121	8.0	593	***
Low															
Practical support	5.3	15	2.4	17	***	9.7	28	2.8	100	***	12.4	49	3.4	251	***
Social support	17.6	50	11.0	78	***	22.0	63	9.2	327	***	20.4	80	9.7	710	***
Trust in others	53.0	151	39.0	277	***	44.8	128	24.0	855	***	31.1	123	14.9	1091	***
Participation in social activities	22.9	66	9.5	68	***	32.3	94	14.2	512	***	37.4	152	20.8	1548	***
Participation in sports events	68.4	197	44.2	317	***	71.8	209	44.1	1592	***	74.4	302	52.2	3891	***
Trust in unemployment benefits	81.8	198	81.8	494		80.1	217	77.2	2564		75.5	284	76.3	4738	
Trust in health care systems	42.5	108	37.3	248		47.8	133	36.5	1284	***	41.5	160	31.1	2246	***

Pearson chi-square was applied between unemployed and employed for each determinant of health. *** denotes significance at the 5% level

### Health gap between employed and unemployed

In order to identify to what extent social determinants could explain the health gap, the first step was to investigate whether there were difference in health depending on employment. Psychological distress was found to be significantly more common among unemployed than employed in all age groups and genders (Tables 2, 3, and 4). The smallest difference in health between employed and unemployed were found among young men (0.119, *p* < 0.05), followed by middle-aged men (0.127, *p* < 0.05) and women (0.144, *p* < 0.05). The largest health gap was found among young women (0.197, *p* < 0.05), adult men (0.184 *p* < 0.05) and women (0.167, *p* < 0.05).

**Table 2:** Youth (age 16 to 25) – Decomposition of the mental health inequalities between employed and unemployed

	Men			Women		
	Estimate		Share	Estimate		Share
	*Log odds*		*(%)*	*Log odds*		*(%)*
Health gap						
GHQ unemployed	.200			.421		
GHQ employed	.081			.224		
Difference in GHQ	.119	***		.197	***	
*Explained*						
*Socioeconomic background*						
Education	−.030		−25%	.011		6%
Civil status	.014		11%	.007		3%
Occupational class	−.008		−7%	.004		2%
*Economic factors*						
Income^a^	−.007		−6%	.002		1%
Cash margin	.052	***	43%	.030		15%
Financial strain	.010		8%	.024		12%
*Social network*						
Practical support	.001		1%	.021		11%
Trust in others	.019		16%	.008		4%
Social support	.010		9%	.003		2%
Socially active	−.020		−17%	.000		0%
Sports events	−.008		−6%	−.006		−3%
*Macroeconomic trust*						
Unemployment benefits	.001		0%	−.001		0%
Health care	.018		15%	−.001		−1%
*Total*	.052		43%	.102		51%
Unexplained	.068		57%	.096		49%

^a^Reference category; lowest income quintile

*** denotes significance at the 5% level. 2. Share is the percentage of the predicted contribution for each independent variable in the health gap between employed and unemployed

**Table 3:** Adulthood (age 26 to 39) – Decomposition of the mental health inequalities between employed and unemployed

	Men			Women		
	Estimate		Share	Estimate		Share
	*Log odds*		*(%)*	*Log odds*		*(%)*
Health gap						
GHQ unemployed	.305			.350		
GHQ employed	.120			.183		
Difference in GHQ	.184	***		.167	***	
*Explained*						
*Socioeconomic background*						
Education	.007		4%	−.004		−3%
Civil status	.018		10%	−.003		−2%
Occupational class^a^	−.008		−4%	−.044	***	−27%
*Economic factors*						
Income	.039	***	21%	−.005		−3%
Cash margin	.025		13%	.028	***	16%
Financial strain	.046	***	25%	.045	***	27%
*Social network*						
Practical support	−.002		−1%	.008		5%
Trust in others	.021	***	11%	.016	***	10%
Social support	.011		6%	.014	***	8%
Socially active	.003		2%	.000		0%
Sports events	−.001		−1%	.008		5%
*Macroeconomic trust*						
Unemployment benefits	.001		0%	.000		0%
Health care	.009		5%	.008		5%
*Total*	.169		92%	.070		42%
Unexplained	.016		8%	.097		58%

^a^Reference category; lowest income quintile

*** denotes significance at the 5% level. 2. Share is the percentage of the predicted contribution for each independent variable in the health gap between employed and unemployed

**Table 4:** Middle age (age 40 to 65) – Decomposition of the mental health inequalities between employed and unemployed

	Men			Women		
	Estimate		Share	Estimate		Share
	*Log odds*		*(%)*	*Log odds*		*(%)*
Health gap						
GHQ unemployed	.217			.286		
GHQ employed	.090			.142		
Difference in GHQ	.127	***		.144	***	
*Explained*						
*Socioeconomic background*						
Education	−.008	***	−7%	−.018	***	−13%
Civil status	−.008		−7%	−.007		−5%
Occupational class^a^	−.020	***	−15%	−.008		−5%
*Economic factors*						
Income	.022		17%	.003		2%
Cash margin	.024	***	19%	.015		10%
Financial strain	.018	***	15%	.036	***	25%
*Social network*						
Practical support	.011	***	9%	.009		6%
Trust in others	.017	***	13%	.028	***	19%
Social support	.020	***	15%	.006		4%
Socially active	.004		3%	.003		2%
Sports events	−.001		−1%	.009	***	7%
*Institutional trust*						
Unemployment benefits	−.002		−1%	.000		0%
Health care	.001		1%	.017	***	12%
*Total*	.077		60%	.093		65%
Unexplained	.050		40%	.051		35%

^a^Reference category; lowest income quintile

*** denotes significance at the 5% level. 2. Share is the percentage of the predicted contribution for each independent variable in the health gap between employed and unemployed

### Decomposing the health gap

The second step was to decompose the health gap, reported for each age group in Tables 2, 3, and 4. In the age group of 16 to 25 (Table 2), the explained proportion of the health inequality were 43 and 51% among men and women, respectively, attributed to the social determinants of health between unemployed and employed. Among men, the single most contributing factor was cash margin, which explained 43% of the health gap (*p* < 0.05). In addition, experience of trust in others and trust in health care (15–16%) showed to be contributing factors, but not significant, among young men. Among young women, none of the single predictors showed a statistical significant contribution. However, cash margin (15%), financial strain (12%) and practical support (11%) were the most relevant.

In adulthood (26 to 39 years old), the social determinants of health explained 92% of the health gap among men and 42% of the health gap among women (Table 3). Financial strain showed to be the single most contributing factors for both sexes (25 and 27% respectively, *p* < 0.05). Among adult men, the significant contributors to the health gap were income (21%) and trust in others (11%), while the corresponding factors among women were cash margin (16%) trust in others (10%), and social support (8%). A negative contributing factor among adult women was occupational class (− 27%, *p* < 0.05). This implies that among women in the age group of 26 to 39 years old, employed were more often white-collar workers than unemployed, and this, in combination with white-collar work being associated to poor mental health among unemployed adult women leading to increase, rather than decrease, in health inequality between the groups.

The social determinants of health in middle age (age 40 to 65) explained 60 and 65% of the health inequality between employment status among men and women, respectively (Table 4). Similar to previous age groups, the economic factors showed to be the main contributing factors; cash margin (19%) and financial strain (15%) among men, while financial strain (25%) among women (*p* < 0.05). Other key significant determinants of health were social support (15%), trust in others (13%) and practical support (9%) among men, and trust in others (19%), trust in health care (12%), participation in sport events (7%) among women. In middle age, education showed to be a factor increasing the health gap between employed and unemployed (− 7% among men, − 13% among women, *p* < 0.05). Among men, occupational class also showed to be negatively contributing to the health inequality (− 15%, *p* < 0.05).

Overall, socioeconomic background did not have a key role in explaining the health inequality. Having low education, not cohabiting and blue-collar occupational class were characteristics more common among those in unemployment, emphasising the uneven distribution of socioeconomic resources between employments.

## Discussion

In this study, we analysed the distribution of psychological distress between employed and unemployed in northern Sweden, and decomposed the health inequalities by domains such as socioeconomic status, access to economic resources, participation in social networks and trust in the institutional system. This is, to the best of our knowledge, the first study showing how key determinants contribute to explaining the health gap between employed and unemployed, and the way in which they differently influence across age and gender.

Results from this study correspond to previous findings in the field [3, 5, 10, 58], showing inequalities in health between employed and unemployed, though in our case across age and gender. The smallest difference in health gap in health was found among young men while the largest difference was found among young women. In adulthood and middle age the pattern of health gap was similar across gender. Decomposing the health inequality showed that 43–51% of the total inequality among youths, 42–92% among adults and 60–65% among middle age was mainly attributed to different access to economic and social resources, and to a smaller extent to the trust in the institutional systems.

Employment is one of the most fundamental social determinants of health and life chances, where paid work is the primary tool for the re-distribution of economic wealth in society [20, 27]. In previous national and international research, cash margin and financial strain have been identified as potential mediators in the relationship between unemployment and poor quality of life in northern Sweden [28] and in another study on self-rated health covering 28 European countries [25]. Our findings, as expected, underline that access to economic resources (income, cash margin and financial strain) are crucial factors, across age groups and gender, to reduce the health inequality between unemployed and employed. However, in our study the importance of each economic factor differed across age and gender. For example, for young and middle-aged men the uncertainty of not having a cash margin was a major explanatory factor, while the difficulties managing ongoing expenses (financial strain) showed to be an important factor among adults and middle-aged women. Income was only a significant factor for men in adulthood. This sheds light on how economic stress may operate differently depending on age in combination with labour market position, but commonly influencing mental ill-health among those in unemployment.

This study also recognised how social support and social relations make an important explanatory contribution to mental health inequality. Although our data do not allow an investigation of how social networks may reinforce the ability to keep a job or be re-hired, it paints a picture of the different circumstances that may be related to mental health in the population. The role of social network increased with age by 3–14% in youth, 17–28% in adulthood and 38–39% in middle age. On an individual level, the feeling of belonging to a social network makes people feel cared for, self-esteemed and valued [26], while on a societal level it contributes to mutual obligations and respect in communities, e.g. the social cohesion in a society [59] probably leading to a better mental health. In line with previous research showing associations between lack of social support and lower well-being, depression and mortality [4, 28], our results suggest that trust in others, social and practical support may serve to buffer the psychological distress, which may embed the feeling of self-worth and good life prospects during unemployment. The increasing explanatory impact of social network across the age groups implies that social relations become more important for health across the life course, which can be illustrated by the importance of the *social norm of employment* in adult life. However, another dimension of social network is the ability to handle the loss of working identity. Our findings showed no support for the buffering effect of alternative role identity beyond the work identity.

Institutional trust has been identified as a predictor of social inequality and a key element in the Swedish welfare system [60]. In public health trust is commonly used as a structural component in social capital affecting heath [61, 62] and as an important component for patients seeking health care [63]. In this study, we hypothesised that institutional trust may act as a buffering effect between prospects for the future and the ability to get institutional support in case of unemployment and mental health. However, our results showed limited support for the health-buffering effect attributed to the trust in institutional systems. The general low trust in unemployment benefits, regardless of employment, age and gender, may be seen in the light of the cuts made in the state-financed unemployment benefits and the general downsizing of the social security system in the Swedish welfare system in recent years [9–11]. In contrast, access to unemployment benefits as a mediator for avoiding negative health effects has also shown to be limited in other studies [4, 25]. Trust in the health care system was more evenly distributed among employed and unemployed and between age groups, and was also shown to explain some health inequality, particularly among middle-aged women. The latter finding emphasises the need to further investigate the potential mediating role of health care trust as a way to decrease mental health inequality.

In line with previous research [46, 64], our findings also stress the compositional effect of socioeconomic factors where the unemployed tend to be lower educated, not cohabit with a partner and blue-collar occupational class compared to the employed, with the implication of a modest or negative contribution explaining the health inequality. This may be viewed as a potential selection effect into unemployment, and thus, that the Swedish welfare efforts to reduce the social and material inequity between citizens fails by key social determinants of health [11, 19]. Altogether, this study shed light on how economic stress and access to social networks operated differently depending on the social position of employed and unemployed across three life phases (youth, adulthood and midlife). This implies, for instance, that a targeting policy approach from the government of increased cash margin may be most beneficial among young men while financial strain could be more beneficial among adults and women in midlife in order to reduce labour market health differences.

### Methodological considerations

The limitations of the study need to be addressed. First, with a response rate of 50%, the non-response could be an issue regarding representatively to a larger population. The sensitivity analysis performed by Statistics Sweden showed that the majority of dropouts were registered as not accessible while only a small part were mail returns, declining participation, questionnaire problems or unable to contact. Regarding labour market position, the dropout was only 1.4%, although we were unable to know whether unemployment was a reason for not participating in the survey. Internal dropout due to missing values in one or several explanatory variables resulted in a relatively large share of missing individuals in the analysis, particularly variables such as financial strain, cash margin and support. However, as mentioned in the methods section, no systematic pattern was found in the missing values. Moreover, the cross-sectional study design could not account for previous health status. The health selection may be troubling given the two-way causal direction of unemployment and health [65]. A further limitation is that the study design also limited our ability to account for the complexity of different types of unemployment and unemployment durations, and thus, the actual social and economic change. Earlier research shows that the effect of unemployment on health varies depending on timing, duration and characteristics of the unemployment situation, factors that we did not have the possibility to measure [4, 66]. Lastly, our model hypothesised that unemployed as compared to employed were at higher risk of social and economic exclusion, which in turn is damaging to health. However, unemployment, particularly during shorter periods of time, may also be a health-improving life event, due to poor work environment (such as stress and conflicts), less conflicts related to the work-home balance and fear of unemployment. This heterogeneity within the group of unemployed could not be taken into account due to the study design, which might result in smaller health gap estimations between the employed and unemployed. Different operationalisations of unemployment (including outside the labour market and labour market measure) and employment (including all excluded groups such as those in parental leave, students and sick-leave) were tested, with similar results supporting the validity of our results.

## Conclusions

In this study, mental health inequality between unemployed and employed was found in three age groups and among both women and men. Furthermore, this study identified key social determinants of health inequality, shedding light on how economic stress and access to social networks operated differently depending on the social position across three life phases. Of the total health inequality, 43–51% among youths, 42–92% among adults and 60–65% among middle age was explained by difference in access to economic and social resources, and to a smaller extent by the trust in the institutional systems. These findings reinforce the heterogeneity within unemployed across the life course, and confirm that much of the health inequality in the Swedish labour market is socially and politically produced and potentially avoidable. In a policy perspective, our findings underline that the beneficial effect of public health and labour market policies may vary depending on the individual and contextual characteristics of the target group. To prevent mental health inequality, future research and public health work should give greater consideration to the social and economic deprivation among unemployment considering a life course perspective.

## References

[1] Lahelma E, Kivela K, Roos E, Tuominen T, Dahl E, Diderichsen F, Elstad JI, Lissau I, Lundberg O, Rahkonen O (2002). Analysing changes of health inequalities in the Nordic welfare states. Soc Sci Med.

[2] Buffel V, van de Straat V, Bracke P (2015). Employment status and mental health care use in times of economic contraction: a repeated cross-sectional study in Europe, using a three-level model. Int J Equity Health.

[3] Virtanen P, Liukkonen V, Vahtera J, Kivimaki M, Koskenvuo M (2003). Health inequalities in the workforce: the labour market core-periphery structure. Int J Epidemiol.

[4] McKee-Ryan FM, Song ZL, Wanberg CR, Kinicki AJ (2005). Psychological and physical well-being during unemployment: a meta-analytic study. J Appl Psychol.

[5] Paul KI, Moser K (2009). Unemployment impairs mental health: meta-analyses. J Vocat Behav.

[6] Brydsten A, Hammarstrom A, Strandh M, Johansson K. Youth unemployment and functional somatic symptoms in adulthood: results from the northern Swedish cohort. Eur J Pub Health. 2015;25(5):796-800. 10.1093/eurpub/ckv038 25772751

[7] Strandh M, Winefield A, Nilsson K, Hammarstrom A (2014). Unemployment and mental health scarring during the life course. Eur J Pub Health.

[8] Hammarstrom A, Janlert U (2002). Early unemployment can contribute to adult health problems: results from a longitudinal study of school leavers. J Epidemiol Community Health.

[9] Farrants K, Bambra C, Nylen L, Kasim A, Burstrom B, Hunter D (2016). Recommodification, Unemployment, and health inequalities: trends in England and Sweden 1991-2011. Int J Health Ser: Plann Administration Eval.

[10] Blomqvist S, Burstrom B, Backhans MC (2014). Increasing health inequalities between women in and out of work--the impact of recession or policy change? A repeated cross-sectional study in Stockholm county, 2006 and 2010. Int J Equity Health.

[11] Raphael D (2014). Challenges to promoting health in the modern welfare state: the case of the Nordic nations. Scand J Public Health.

[12] Sweden S: Arbetsmarknadssituationen för hela befolkningen 15–74 år. In.; 2014.

[13] Arbetskraftsundersökningarna: Utvecklingen på den svenska arbetsmarknaden sedan finanskrisen [Development of the Swedish labour market since the financial crisis]. In*.*; 2014.

[14] Esping-Andersen G (1990). The three worlds of welfare capitalism: Cambridge: polity.

[15] Marmot M, Friel S, Bell R, Houweling TAJ, Taylor S (2008). Closing the gap in a generation: health equity through action on the social determinants of health. Lancet.

[16] Barnen R: Barnfattigdom i Sverige–årsrapport 2010. Tillgänglig: www raddabarnen se 2010.

[17] Almqvist A (2016). Den ekonomiska ojämlikheten i Sverige. Landsorganisationen i Sverige.

[18] Marmot M, Allen J, Bell R, Bloomer E, Goldblatt P (2012). WHO European review of social determinants of health and the health divide. Lancet.

[19] Lundberg O, Yngwe MÅ, Stjärne MK, Elstad JI, Ferrarini T, Kangas O, Norström T, Palme J, Fritzell J (2008). The role of welfare state principles and generosity in social policy programmes for public health: an international comparative study. Lancet.

[20] Bambra C (2012). Work, worklessness, and the political economy of health: Oxford university press.

[21] Bartley M (1994). Unemployment and ill health: understanding the relationship. J Epidemiol Community Health.

[22] van der Noordt M, IJ H, Droomers M, Proper KI (2014). Health effects of employment: a systematic review of prospective studies. Occup Environ Med.

[23] McLeod CB, Hall PA, Siddiqi A, Hertzman C. How society shapes the health gradient: work-related health inequalities in a comparative perspective. Annu Rev Public Health. 2012;33 33:59−+ 10.1146/annurev-publhealth-031811-124603 22429159

[24] Strandh M (2000). Different exit routes from unemployment and their impact on mental well-being: the role of the economic situation and the predictability of the life course. Work Employ Soc.

[25] Toge AG (2016). Health effects of unemployment in Europe (2008-2011): a longitudinal analysis of income and financial strain as mediating factors. Int J Equity Health.

[26] Jahoda M (1981). Work, employment, and unemployment - values, theories, and approaches in social-research. Am Psychol.

[27] Weeks K (2011). The problem with work: feminism, Marxism, antiwork politics, and postwork imaginaries.

[28] Hultman B, Hemlin S, Hornquist JO (2006). Quality of life among unemployed and employed people in northern Sweden. Are there any differences?. Work.

[29] Hammer T (2000). Mental health and social exclusion among unemployed youth in Scandinavia. A comparative study. Int J Soc Welf.

[30] Andersson K (2005). Ideal och verklighet.: Studiecirkelns betydelse för lärande och hälsa.

[31] Beckfield J, Krieger N (2009). Epi + demos + cracy: linking political systems and priorities to the magnitude of health inequities--evidence, gaps, and a research agenda. Epidemiol Rev.

[32] Burstrom B, Nylen L, Barr B, Clayton S, Holland P, Whitehead M (2012). Delayed and differential effects of the economic crisis in Sweden in the 1990s on health-related exclusion from the labour market: a health equity assessment. Soc Sci Med.

[33] O'Campo P, Molnar A, Ng E, Renahy E, Mitchell C, Shankardass K, St John A, Bambra C, Muntaner C (2015). Social welfare matters: a realist review of when, how, and why unemployment insurance impacts poverty and health. Soc Sci Med.

[34] Vahid Shahidi F, Siddiqi A, Muntaner C. Does social policy moderate the impact of unemployment on health? A multilevel analysis of 23 welfare states. Eur J Pub Health. 2016;26(6):1017-1022. 10.1093/eurpub/ckw050 27060593

[35] Niedzwiedz CL, Mitchell RJ, Shortt NK, Pearce JR (2016). Social protection spending and inequalities in depressive symptoms across Europe. Soc Psychiatry Psychiatr Epidemiol.

[36] van Rijn RM, Carlier BE, Schuring M, Burdorf A (2016). Work as treatment? The effectiveness of re-employment programmes for unemployed persons with severe mental health problems on health and quality of life: a systematic review and meta-analysis. Occup Environ Med.

[37] Knabe A, Rätzel S (2010). Better an insecure job than no job at all? Unemployment, job insecurity and subjective wellbeing. Econ Bull.

[38] Nordenmark M, Strandh M, Layte R (2006). The impact of unemployment benefit system on the mental well-being of the unemployed in Sweden, Ireland and great Britain. Eur Soc.

[39] Green L (2014). Understanding the life course: sociological and psychological perspectives: John Wiley & Sons.

[40] Larkin M (2013). Health and well-being across the life course: sage.

[41] Hayford SR, Furstenberg FF (2008). Delayed adulthood, delayed desistance? Trends in the age distribution of problem behaviors. J Res Adolesc.

[42] Doiron D, Mendolia S (2012). The impact of job loss on family dissolution. J Popul Econ.

[43] Worach-Kardas H, Kostrzewski S (2014). Quality of life and health state of long - term unemployed in older production age. Appl Res Qual Life.

[44] Vansteenkiste S, Deschacht N, Sels L (2015). Why are unemployed aged fifty and over less likely to find a job? A decomposition analysis. J Vocat Behav.

[45] Wagenaar AF, Kompier MAJ, Houtman ILD, van den Bossche SNJ, Taris TW (2015). Who gets fired, who gets re-hired: the role of workers' contract, age, health, work ability, performance, work satisfaction and employee investments. Int Arch Occup Environ Health.

[46] Puig-Barrachina V, Malmusi D, Martenez JM, Benach J (2011). Monitoring social determinants of health inequalities: the impact of unemployment among vulnerable groups. Int J Health Ser: Plann Administration Eval.

[47] Marmot M (2005). Social determinants of health inequalities. Lancet.

[48] Public Health Agency of Sweden: Hälsa på lika villkor [Health on Equal Terms]. In*.*; 2014.

[49] Lundin A, Hallgren M, Theobald H, Hellgren C, Torgén M (2016). Validity of the 12-item version of the general health questionnaire in detecting depression in the general population. Public Health.

[50] Sweden S (1984). Socio-economic classification [Socioekonomisk indelning (SEI)]: SCB.

[51] Yun M-S (2004). Decomposing differences in the first moment. Econ Lett.

[52] Yun M-S (2005). Hypothesis tests when decomposing differences in the first moment. J Econ Soc Meas.

[53] Oaxaca R. Male-female wage differentials in urban labor markets. Int Econ Rev. 1973:693–709.

[54] San Sebastian M, Hammarstrom A, Gustafsson PE (2015). Socioeconomic inequalities in functional somatic symptoms by social and material conditions at four life course periods in Sweden: a decomposition analysis. BMJ Open.

[55] Hosseinpoor AR, Stewart Williams J, Amin A, Araujo de Carvalho I, Beard J, Boerma T, Kowal P, Naidoo N, Chatterji S (2012). Social determinants of self-reported health in women and men: understanding the role of gender in population health. PLoS One.

[56] Jann B, Stata A (2008). Implementation of the blinder-Oaxaca decomposition. Stata J.

[57] Fairlie RW (2005). An extension of the blinder-Oaxaca decomposition technique to logit and probit models. J Econ Soc Meas.

[58] Janlert U, Hammarstrom A (2009). Which theory is best? Explanatory models of the relationship between unemployment and health. BMC Public Health.

[59] Stansfeld SA, Marmot M, Wilkinson R (2006). Social support and social cohesion. Soc Determinants Health.

[60] Bäckström A, Edlund J (2012). Understanding the link between trust in public institutions and welfare policy preferences.

[61] Moore S, Kawachi I. Twenty years of social capital and health research: a glossary. J Epidemiol Community Health. 2017:jech-2016–208313. 10.1136/jech-2016-208313 28087811

[62] Rostila M (2007). Social capital and health in European welfare regimes: a multilevel approach. J Eur Soc Policy.

[63] Mohseni M, Lindstrom M (2007). Social capital, trust in the health-care system and self-rated health: the role of access to health care in a population-based study. Soc Sci Med.

[64] Backhans MC, Hemmingsson T (2012). Unemployment and mental health--who is (not) affected?. Eur J Pub Health.

[65] Janlert U (1997). Unemployment as a disease and diseases of the unemployed. Scand J Work Env Hea.

[66] Janlert U, Winefield AH, Hammarstrom A (2015). Length of unemployment and health-related outcomes: a life-course analysis. Eur J Pub Health.

